# Alterations in protein and amino acid metabolism in rats fed a branched-chain amino acid- or leucine-enriched diet during postprandial and postabsorptive states

**DOI:** 10.1186/s12986-016-0072-3

**Published:** 2016-02-11

**Authors:** Milan Holecek, Pavel Siman, Melita Vodenicarovova, Roman Kandar

**Affiliations:** Department of Physiology, Faculty of Medicine in Hradec Kralove, Charles University in Prague, Simkova 870, Hradec Kralove, 500 38 Czech Republic; Department of Biochemistry, Faculty of Medicine in Hradec Kralove, Charles University Prague, Hradec Kralove, Czech Republic; Department of Biological and Biochemical Sciences, Faculty of Chemical Technology, University of Pardubice, Pardubice, Czech Republic

**Keywords:** Nutritional supplements, Glutamine, Protein synthesis, Proteolysis, Muscle, Starvation

## Abstract

**Background:**

Many people believe in favourable effects of branched-chain amino acids (BCAAs; valine, leucine, and isoleucine), especially leucine, on muscle protein balance and consume BCAAs for many years. We determined the effects of the chronic intake of a BCAA- or leucine-enriched diet on protein and amino acid metabolism in fed and postabsorptive states.

**Methods:**

Rats were fed a standard diet, a diet with a high content of valine, leucine, and isoleucine (HVLID), or a high content of leucine (HLD) for 2 months. Half of the animals in each group were sacrificed in the fed state on the last day, and the other half were sacrificed after overnight fast. Protein synthesis was assessed using the flooding dose method (L-[3,4,5-^3^H]phenylalanine), proteolysis on the basis of chymotrypsin-like activity (CHTLA) of proteasome and cathepsin B and L activities.

**Results:**

Chronic intake of HVLID or HLD enhanced plasma levels of urea, alanine and glutamine. HVLID also increased levels of all three BCAA and branched-chain keto acids (BCKA), HLD increased leucine, ketoisocaproate and alanine aminotransferase and decreased valine, ketovaline, isoleucine, ketoisoleucine, and LDL cholesterol. Tissue weight and protein content were lower in extensor digitorum longus muscles in the HLD group and higher in kidneys in the HVLID and HLD groups. Muscle protein synthesis in postprandial state was higher in the HVLID group, and CHTLA was lower in muscles of the HVLID and HLD groups compared to controls. Overnight starvation enhanced alanine aminotransferase activity in muscles, and decreased protein synthesis in gastrocnemius (in HVLID group) and extensor digitorum longus (in HLD group) muscles more than in controls. Effect of HVLID and HLD on CHTLA in muscles in postabsorptive state was insignificant.

**Conclusions:**

The results failed to demonstrate positive effects of the chronic consumption of a BCAA-enriched diet on protein balance in skeletal muscle and indicate rather negative effects from a leucine-enriched diet. The primary effects of both diets are an activated catabolism of BCAAs, which leads to an enhanced production of BCKA, alanine and glutamine and their utilization in visceral tissues and an impaired protein synthesis in postabsorptive state, particularly in fast-twitch (white) muscles.

## Background

The branched-chain amino acids (BCAAs) valine, leucine and isoleucine are essential substrates and important regulators in the synthesis of body proteins, substrates for energy production and precursors for the formation of other amino acids. The stimulatory effect of BCAAs on protein synthesis and the inhibitory effect on proteolysis have been known for many years [1–3]. Particularly, leucine was implicated in the stimulation of protein synthesis in skeletal muscle. Leucine enhances insulin release from β-cells of the pancreas and directly stimulates protein synthesis through the mammalian target of rapamycin (mTOR) signalling pathway and the phosphorylation of translation initiation factors and ribosomal proteins [4, 5]. The inhibitory effect of BCAAs on proteolysis is likely mediated by several metabolites of BCAAs, particularly branched-chain keto acids and beta-hydroxy-beta-methylbutyrate [6, 7].

**Fig. 1:**
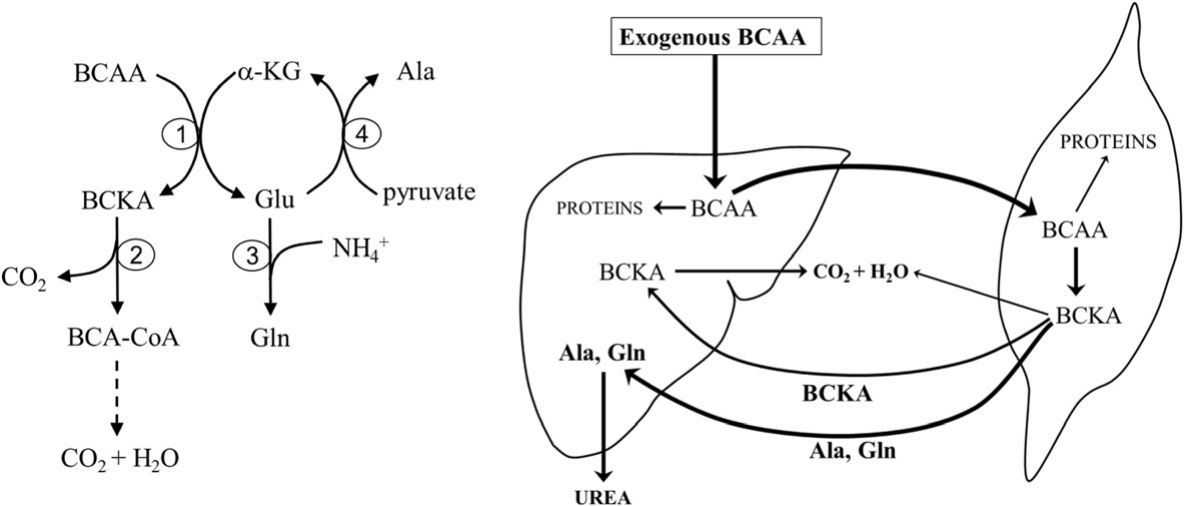
The primary pathways of BCAA catabolism in skeletal muscle and the supposed response of skeletal muscle and liver to surplus exogenous BCAAs. 1, BCAA aminotransferase; 2, BCKA dehydrogenase; 3, glutamine synthetase; 4, alanine aminotransferase (ALT)

The initial site for most BCAA catabolism is skeletal muscle because of the high activity of BCAA aminotransferase, which enables the transfer of the amino group of BCAAs to α-ketoglutarate to form glutamate and branched-chain keto acids (BCKA), i.e., α-ketoisocaproate (KIC, ketoleucine), α-keto-β-methylvalerate (KMV, ketoisoleucine) and α-ketoisovalerate (KIV, ketovaline). The enhanced availability of glutamic acid increases the flux through glutamine synthetase and alanine aminotransferase leading to enhanced synthesis of glutamine and alanine. These amino acids are released together with most of the BCKA from skeletal muscle to the blood [8–10]. Overall, an enhanced intake of BCAAs should lead to enhanced BCAA catabolism and the release of glutamine, alanine and BCKA from muscles to the blood stream (Fig. 1).

The unique effects of BCAAs on protein metabolism led to the use of BCAAs in patients with cachectic disorders and as popular dietary supplements, especially in athletes. However, chronic and excessive intake of the BCAAs raises concerns of their adverse side effects. The chronic intake of high amounts of BCAAs may induce an imbalance in amino acid concentrations in body fluids, alter various biochemical pathways and cellular functions, and the response of the body to different physiological and pathological conditions, such as starvation, exercise, trauma, infection, and cancer development. Unfortunately, few studies have investigated the long-term effects of BCAA supplementation, and there is a lack of information on the side effects and consequences of the long-term intake of these supplements.

We determined how the chronic intake of a BCAA- or leucine-enriched diet affected protein and amino acid homeostasis, particularly the effect on protein balance in skeletal muscle and on the concentrations of free amino acids in extracellular and intracellular spaces, especially BCAA, alanine, and glutamine levels. Various types of hindlimb muscles were examined, including m. soleus (SOL, slow-twitch, red muscles), m. extensor digitorum longus (EDL, fast-twitch, white muscle), and m. gastrocnemius (GM, both fibre types), because of reported differences in protein, BCAA and glutamine metabolism in slow-twitch and fast-twitch muscles [11–13].

The effects of BCAA- or leucine-enriched diet have been examined in two nutritionally different conditions—in fed and overnight fasted animals. In fed (postprandial) state are concentrations of nutrients in body fluids closely related to composition of the food and anabolic response of the body mediated by enhanced secretion of insulin. After overnight fast (in postabsorptive state) is the effect of food composition on concentration of nutrients in extracellular fluid smaller and the main role play gradual decrease in insulin/glucagon ratio and enhanced catabolism of glycogen, lipids, and proteins.

## Methods

### Animals and material

Male Wistar rats (BioTest, Konarovice, CR) were housed in standardised cages in quarters with controlled temperature and a 12-h light–dark cycle. All rats received the standard laboratory diet (SLD) ST-1 (Velas, CR) and drinking water *ad libitum*. All procedures involving animals were performed according to the guidelines set by the Institutional Animal Care and Use Committee of Charles University. Animal Care and Use Committee of Charles University in Prague, Faculty of Medicine in Hradec Kralove specifically approved this study. L-[3,4,5-^3^H]phenylalanine was purchased from American Radiolabeled Chemical, Inc. (St. Louis, MO, USA). Chemicals were obtained from Sigma Chemical (St. Louis, MO, USA), Lachema (Brno, CR), Waters (Milford, MA, ISA), Biomol (Hamburg, Germany), and Merck (Darmstadt, Germany).

### Experimental design

A total of 120 male Wistar rats weighing approximately 200 g each were randomly divided into three groups fed an SLD or a diet in which 10 % of the basal diet was replaced by a mixture of valine, leucine, and isoleucine in ratios of 1 : 1 : 1 (HVLID, high valine, leucine, and isoleucine diet) or leucine (HLD, high leucine diet). The mixtures were used to prepare pellets with the same physical properties and consistency. Estimated contents of L-valine, L-leucine, and L-isoleucine (g/kg diet) are shown in Table 1. These contents resemble a high-dose supplementation in which adverse outcomes for the monitored variables were not reported [14–16].

**Table 1:** Estimated contents of L-valine, L-leucine, and L-isoleucine in experimental diets (g/kg diet)

Diet	Valine (g/kg)	Leucine (g/kg)	Isoleucine (g/kg)
SLD	14	21	12
HVLID	46	50	44
HLD	13	120	11

*SLD* standard laboratory diet; *HVLID* diet with high content of valine, leucine, and isoleucine; *HLD* high leucine diet

The animals consumed tested diets for 2 months and were sacrificed between 7 and 8 a.m. on the last day of the study protocol. Half of the animals in each group were sacrificed in the fed state, and the other half were sacrificed after an overnight fast. Two separate studies were performed. Tissue protein synthesis rates were measured using the flooding dose method (L-[3,4,5-^3^H]phenylalanine) in the first study. Alterations in amino acid concentrations in body fluids and various parameters of protein and amino acid metabolism were estimated in the second study.

### Protein synthesis

The rats were injected intravenously with a flooding dose of L-[3,4,5-^3^H]phenylalanine (50 μCi/100 g b.w.) combined with unlabelled L-phenylalanine (150 μmol/100 g b.w.) 10 min before the sacrifice by exsanguination via the abdominal aorta [17]. Small pieces (approximately 0.1 g) of soleus (SOL), gastrocnemius (GM), and extensor digitorum longus (EDL) muscles, liver, kidneys, and jejunum were quickly removed and frozen in liquid nitrogen. Tissue samples were homogenized in 6 % (v/v) perchloric acid, and the precipitated proteins were collected via centrifugation for 5 min at 12,000 g. The supernatant was used for the measurement of L-[3,4,5-^3^H]phenylalanine specific activity. The pellet was washed three times and hydrolysed in 2 N NaOH. Aliquots were taken for protein content [18] and radioactivity measurements. The fractional rate of protein synthesis (FRPS) was calculated according the formula derived by McNurlan et al. [19]:

$$ FRPS\ \left(\%\  per\  day\right) = \left({S}_b\cdot 100\right)/\left(t\cdot {S}_a\right) $$

where *S*_*b*_ and *S*_*a*_ are the specific activities (dpm/nanomole) of protein-bound phenylalanine and tissue-free phenylalanine in the acid-soluble fraction of tissue homogenates, respectively, and *t* is the time (days) between isotope injection and tissue immersion into liquid nitrogen. The value of 274 μmol phenylalanine/g protein was used for the calculation of protein-bound phenylalanine specific activity [20]. Sample radioactivity was measured using a liquid scintillation radioactivity counter LS 6000 (Beckman Instruments, Fullerton, CA, USA).

### Amino acid concentrations in blood plasma and tissues

Amino acid concentrations were determined in the supernatants of deproteinised blood plasma and tissue samples using high-performance liquid chromatography (Aliance 2695, Waters, Milford, MA, USA) after derivatisation with 6-aminoquinolyl-N-hydroxysuccinimidyl carbamate. The intracellular concentration of each amino acid was calculated by subtracting the free extracellular portion from the total amount, assuming the plasma concentration to be equal to the concentration in the interstitial fluid as described by Bergstrőm et al. [21]. Total tissue water was measured from the tissue weight obtained after drying for 24 h at 90 °C. The determination of extra- and intracellular water was based on the chloride method according to Graham et al. [22]. BCKA concentrations in blood plasma were measured using liquid chromatograph (Shimadzu, Kyoto, Japan) after precolumn derivatisation with o-phenylenediamine [23].

### Chymotrypsin-like activity (CHTLA)

The chymotrypsin-like activity of proteasomes was determined using the fluorogenic substrate Suc-LLVY-MCA [24] as follows. The muscles were homogenised in 0.4 ml of ice-cold 20 mM Tris buffer, pH 7.5, containing 2 mM ATP, 5 mM MgCl_2_ and 1 mM dithiothreitol. The homogenates were centrifuged for 10 min at 18,000 g at 4 °C. Cellular supernatants (0.1 ml) were incubated with 0.1 ml of substrate Suc-LLVY-MCA (0.1 mM) with or without inhibitor MG132 (0.02 mM) for 1 h on ice. A volume of 0.4 ml of 100 mM sodium acetate buffer (pH 4.3) was added to stop the reaction. Sample fluorescence was immediately determined at an excitation wavelength of 340 nm and emission wavelength of 440 nm (Tecan Infinite^TM^ 200). The standard curve was established for 7-amino-4-methylcoumarin (AMC), which permitted the expression of CHTLA as nmol of AMC/g protein/hour. The activity was adjusted for the protein concentration of the supernatant. Differences after the subtraction of inhibited from non-inhibited activities were used for calculations.

### Cathepsin B and L activities

The activities of cathepsin B and L were determined using the fluorogenic substrate Z-FA-MCA [25, 26] as follows. Tissue samples (approximately 20 mg) were homogenised in 0.6 ml of ice-cold 300 mM sodium acetate buffer, pH 5.0, containing 4 mM EDTA, 8 mM dithiothreitol and 0.2 % Triton X-100 (v/v). The homogenates were allowed to stand for 30 min on ice and centrifuged for 30 min at 18,000 g at 4 °C. Cellular supernatant (0.01 ml) were incubated with 0.19 ml of substrate Z-FA-MCA (0.1 mM) with or without the inhibitor Z-FF-FMK (0.04 mM) for 30 min at 37 °C. The reaction was stopped by the addition of 1 ml of 100 mM sodium acetate buffer, pH 4.3, and the activities of cathepsin B and L were determined as described above for CHTLA.

### Other techniques

Glutamine synthetase activity was determined using a colorimetric assay based on the formation of γ-glutamyl-hydroxamate [27]. The catalytic concentration of alanine aminotransferase was determined from the rate of decrease of NADH, measured at 340 nm, by lactate dehydrogenase [28]. Enzyme activities are reported per milligram of protein of reaction mixture. Plasma levels of urea, creatinine, ALT, AST, glucose, triglycerides, and cholesterol were measured using commercial tests (Boehringer, Mannheim, Germany; Elitech, Sées, France and Lachema, Brno, CR).

### Statistics

Results are expressed as means ± SE. Analysis of variance (ANOVA) followed by Bonferroni multiple comparison post hoc analysis was used to detect differences between multiple independent groups. NCSS 2001 statistical software (Kaysville, UT, USA) was used for analyses. Differences were considered significant at *P* < 0.05.

## Results

### Effects of HVLID and HLD on food intake, body weight and blood biochemistry

**Fig. 2:**
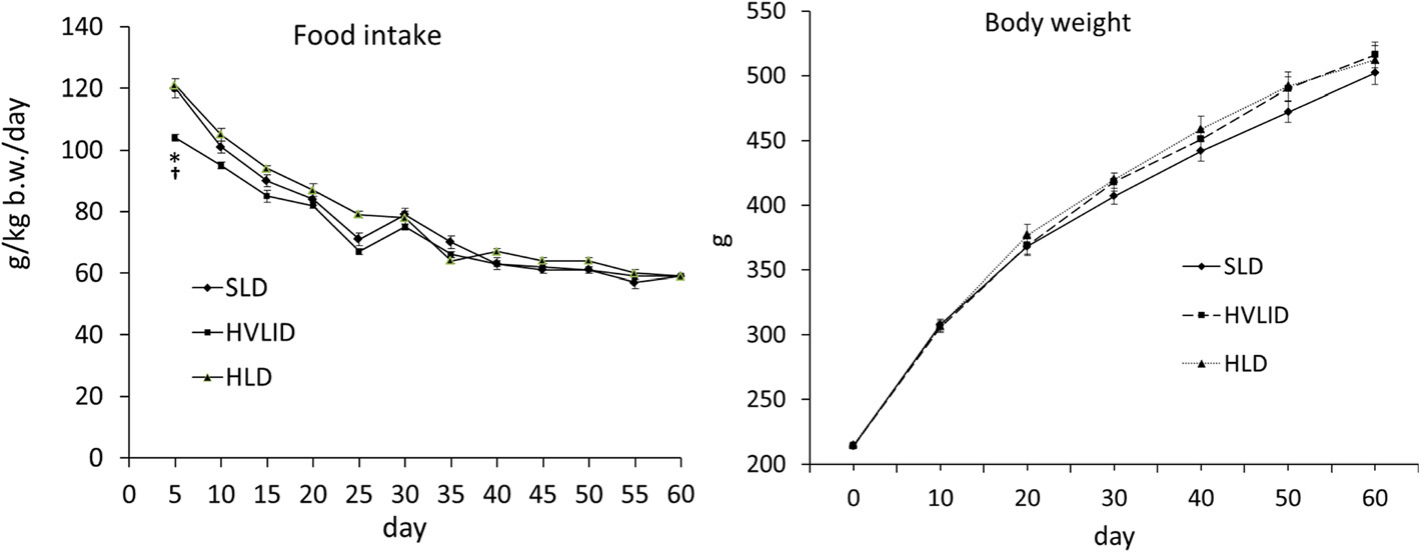
Food intake and body weight changes in rats fed an SLD, HVLID and HLD. Means ± SE, *p* ˂ 0.05. *compared to the SLD group; ^†^ HLD group vs. HVLID group

The intake of HVLID was lower than the intake of SLD and HLD in the initial phase of the study. Differences in daily food intake were not significant beginning the second week. There were no differences in body weight gain between animals receiving the various diets (Fig. 2). Higher blood plasma concentrations of urea were observed in animals that chronically consumed HVLID or HLD. In HLD fed animals we observed higher ALT activity and lower concentration of LDL cholesterol and atherogenicity index than in controls (Table 2).

**Table 2:** Changes in blood plasma

	Fed animals			Overnight starved animals		
	SLD (*n* = 10)	HVLID (*n* = 10)	HLD (*n* = 9)	SLD + S (*n* = 10)	HVLID + S (*n* = 10)	HLD + S (*n* = 10)
Glucose (mmol/l)	9.67 ± 0.21	chylosity	8.58 ± 0.29 ^a^	7.17 ± 0.18 ^b^	8.84 ± 0.28 ^a^	7.96 ± 0.24
Urea (mmol/l)	7.32 ± 0.21	9.60 ± 0.14 ^a^	8.86 ± 0.32 ^a^	6.31 ± 0.25 ^b^	8.23 ± 0.24 ^a,b^	9.00 ± 0.36 ^a^
Creatinine (μmol/l)	27.9 ± 1.0	29.4 ± 1.1	27.6 ± 0.6	30.2 ± 1.3	30.9 ± 1.7	29.9 ± 0.85
ALT (μkat/l)	0.88 ± 0.05	0.97 ± 0.06	1.22 ± 0.03 ^a,c^	0.68 ± 0.04 ^b^	0.59 ± 0.05 ^a^	0.86 ± 0.06 ^a,b,c^
AST (μkat/l	1.35 ± 0.15	1.33 ± 0.05	1.16 ± 0.06	1.26 ± 0.05	1.29 ± 0.06	1.15 ± 0.05
Cholesterol (mmol/l)	1.64 ± 0.11	1.49 ± 0.08	1.54 ± 0.12	1.42 ± 0.10	1.47 ± 0.06	1.30 ± 0.07
HDL cholesterol (mmol/l)	1.08 ± 0.12	0.99 ± 0.04	1.31 ± 0.10 ^c^	1.24 ± 0.09	1.02 ± 0.04	1.16 ± 0.07
LDL cholesterol (mmol/l)	0.29 ± 0.03	chylosity	0.17 ± 0.03 ^a^	0.20 ± 0.03 ^b^	0.28 ± 0.02	0.12 ± 0.01 ^c^
Atherogenicity index	0.39 ± 0.03	0.51 ± 0.05 ^a^	0.19 ± 0.02 ^a,c^	0.14 ± 0.02 ^b^	0.47 ± 0.03 ^a^	0.12 ± 0.02 ^c^
Triglycerides (mmol/l)	1.25 ± 0.07	0.72 ± 0.08 ^a^	0.95 ± 0.06	0.79 ± 0.10 ^b^	0.60 ± 0.07	0.86 ± 0.11
Total protein (g/l)	63.1 ± 0.7	63.0 ± 0.7	64.6 ± 1.2	58.0 ± 0.6 ^b^	61.4 ± 0.8 ^a^	60.3 ± 0.7 ^b^
Albumins (mmol/l)	35.3 ± 1.4	34.7 ± 1.1	35.7 ± 1.8	33.1 ± 0.8	34.8 ± 1.6	33.7 ± 1.2

Means ± SE, *p* ˂ 0.05

*SLD* standard laboratory diet; *SLD + S* rats fed SLD starved overnight before sacrifice; *HVLID* diet with high content of valine, leucine, and isoleucine; *HVLID + S* rats fed HVLID starved overnight before sacrifice; *HLD* high leucine diet; *HLD + S* rats fed HLD starved overnight before sacrifice

^a^ compared to the corresponding control (SLD or SLD + S)

^b^ compared to the corresponding fed group

^c^ HLD (HLD + S) vs. HVLID (HVLID + S). Atherogenicity index was calculated as: (cholesterol-HDL cholesterol)/HDL cholesterol

### Effects of HVLID and HLD on amino acid and BCKA concentrations in blood plasma

**Fig. 3:**
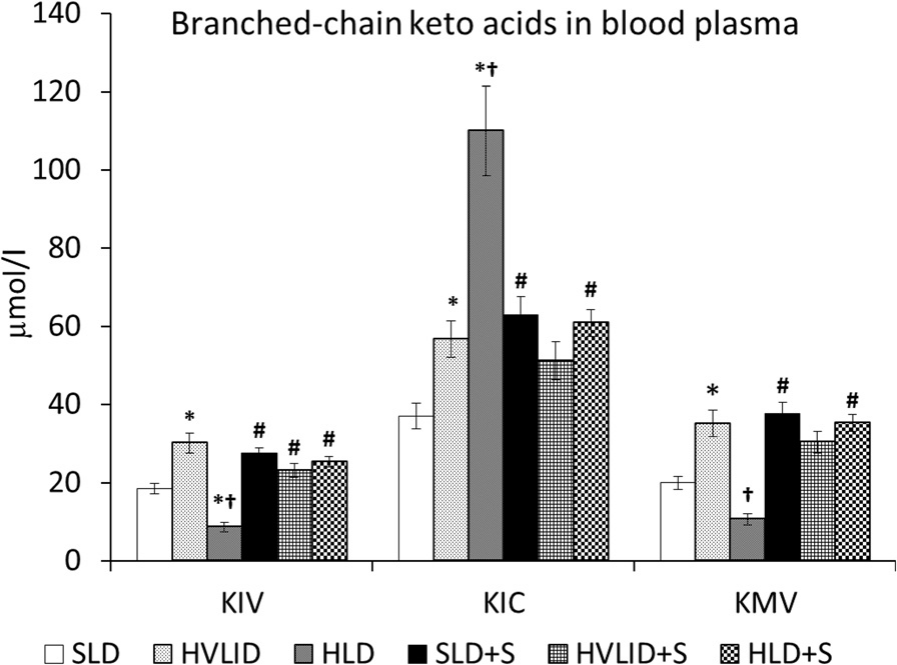
Branched-chain keto acids in blood plasma. Means ± SE, *p* ˂ 0.05. *compared to the corresponding control (SLD or SLD + S); ^#^ compared to the corresponding fed group; ^†^ HLD (HLD + S) group vs. HVLID (HVLID + S) group. KIC: α-ketoisocaproate (ketoleucine), KMV: α-keto-β-methylvalerate (ketoisoleucine), KIV: α-ketoisovalerate (ketovaline)

Plasma concentrations of BCAA, BCKA, alanine, and glutamine increased significantly in animals fed HVLID. Enhanced concentrations of leucine, KIC, glutamine and alanine were also found in animals fed HLD, but concentrations of valine, isoleucine, KIV and KMV were significantly lower than controls. Overnight starvation decreased the plasma concentration of most amino acids in controls and normalised alterations induced by the intake of HVLID or HLD (Table 3 and Fig. 3).

**Table 3:** Amino acid concentrations (μmol/l) in blood plasma

	Fed animals			Overnight starved animals		
	SLD (*n* = 10)	HVLID (*n* = 10)	HLD (*n* = 9)	SLD + S (*n* =10)	HVLID + S (*n* = 10)	HLD + S (*n* = 10)
Asp	17 ± 1	34 ± 3 ^a^	25 ± 3	11 ± 1	26 ± 3 ^a^	24 ± 6 ^a^
Glu	129 ± 8	111 ± 6	108 ± 4	101 ± 6	129 ± 11	128 ± 10
Ser	254 ± 12	239 ± 4	217 ± 6 ^a^	241 ± 8	232 ± 7	236 ± 7
Asn	70 ± 4	78 ± 2	64 ± 2 ^c^	59 ± 2 ^b^	67 ± 2 ^b^	64 ± 2
Gly	306 ± 23	219 ± 6 ^a^	191 ± 10 ^a^	392 ± 15 ^b^	319 ± 12 ^a,b^	281 ± 10 ^a,b^
Gln	713 ± 25	825 ± 12 ^a^	808 ± 19 ^a^	623 ± 10 ^b^	678 ± 16 ^b^	690 ± 14 ^a,b^
His	66 ± 3	65 ± 2	57 ± 1 ^a,c^	54 ± 2 ^b^	56 ± 1 ^b^	54 ± 2
Tau	324 ± 16	467 ± 50	437 ± 52	227 ± 17	528 ± 57 ^a^	520 ± 31 ^a^
Thr	284 ± 17	206 ± 6 ^a^	206 ± 5 ^a^	232 ± 14 ^b^	230 ± 7	226 ± 6
Ctr	82 ± 4	87 ± 3	79 ± 3	60 ± 2 ^b^	74 ± 2 ^a,b^	69 ± 2
Ala	527 ± 27	667 ± 25 ^a^	627 ± 13 ^a^	364 ± 16 ^b^	379 ± 12 ^b^	412 ± 14 ^b^
Arg	188 ± 9	175 ± 5	165 ± 6 ^a^	150 ± 3 ^b^	165 ± 5	157 ± 4
Pro	262 ± 16	251 ± 7	244 ± 6	131 ± 5 ^b^	147 ± 3 ^b^	140 ± 3 ^b^
Tyr	94 ± 15	77 ± 3 ^a^	71 ± 2 ^a^	89 ± 3	85 ± 3	83 ± 3 ^b^
Cys	123 ± 5	122 ± 2	106 ± 3	123 ± 3	138 ± 5	126 ± 5 ^b^
Val	262 ± 8	444 ± 30 ^a^	158 ± 6 ^a,c^	213 ± 7	213 ± 4 ^b^	206 ± 6
Met	63 ± 3	63 ± 1	56 ± 1 ^a^	55 ± 1 ^b^	52 ± 1 ^b^	52 ± 1
Orn	67 ± 3	68 ± 5	69 ± 3	44 ± 2 ^b^	52 ± 2 ^b^	48 ± 2 ^b^
Lys	339 ± 5	332 ± 9	324 ± 13	334 ± 9	417 ± 13 ^b^	440 ± 11 ^b^
Ile	135 ± 19	227 ± 21 ^a^	91 ± 9 ^a,c^	122 ± 6	122 ± 2 ^b^	134 ± 9 ^b^
Leu	228 ± 18	317 ± 21 ^a^	543 ± 35 ^a.c^	177 ± 7	195 ± 4 ^b^	189 ± 6 ^b^
Phe	72 ± 3	71 ± 1	73 ± 1	69 ± 2	72 ± 2	70 ± 2
BCAA	625 ± 37	988 ± 71 ^a^	793 ± 40 ^a,c^	513 ± 20	530 ± 9 ^b^	529 ± 20 ^b^
∑ - BCAA	3975 ± 151	4157 ± 87	3928 ± 94	3361 ± 86 ^b^	3846 ± 75 ^a^	3819 ± 74 ^a^

Means ± SE, *p* ˂ 0.05

^a^ compared to the corresponding control (SLD or SLD + S)

^b^ compared to the corresponding fed group

^c^ HLD (HLD + S) vs. HVLID (HVLID + S). Abbreviations as indicated in Table 1

### Effects of HVLID and HLD on amino acid concentrations in tissues

HVLID increased levels of valine, leucine, and isoleucine and HLD increased leucine and decreased valine and isoleucine concentrations in all muscle types (Table 4). In other tissues the effects of HVLID and HLD were less significant (Table 5). Alanine concentrations were higher in all muscle types of animals that consumed HVLID or HLD and in the jejunum and kidneys of animals that consumed HLD. Glutamine concentrations increased significantly only in muscles of the HLD group. Most of the alterations in intracellular amino acid concentrations that were induced by the chronic intake of HVLID or HLD in the postprandial state disappeared after an overnight fast.

**Table 4:** Amino acid concentrations in muscles (μmol/l of intracellular water)

	Fed animals			Overnight starved animals		
	SLD (*n* = 10)	HVLID (*n* = 10)	HLD (*n* = 9)	SLD + S (*n* =10)	HVLID + S (*n* = 10)	HLD + S (*n* = 10)
*M. gastrocnemius*						
Valine	269 ± 15	547 ± 28 ^a^	166 ± 8 ^a,c^	317 ± 9	352 ± 10 ^b^	336 ± 19 ^b^
Isoleucine	129 ± 10	227 ± 12 ^a^	72 ± 5 ^a,c^	182 ± 5 ^b^	199 ± 5	181 ± 8 ^b^
Leucine	193 ± 13	328 ± 19 ^a^	533 ± 30 ^a,c^	264 ± 7 ^b^	302 ± 10	304 ± 18 ^b^
Glutamine	5773 ± 244	5731 ± 212	6665 ± 236 ^a,c^	3870 ± 141 ^b^	3892 ± 148 ^b^	4714 ± 103 ^a,b,c^
Alanine	3188 ± 165	4838 ± 644^a^	4879 ± 183 ^a^	3227 ± 103	3025 ± 61 ^b^	3467 ± 115 ^b^
*M. soleus*						
Valine	190 ± 8	452 ± 26 ^a^	128 ± 9 ^a,c^	229 ± 9	226 ± 6 ^b^	215 ± 11 ^b^
Isoleucine	92 ± 6	184 ± 12 ^a^	49 ± 6 ^a,c^	126 ± 5 ^b^	116 ± 3 ^b^	109 ± 6 ^b^
Leucine	159 ± 10	287 ± 15	595 ± 93 ^a,c^	231 ± 19	196 ± 4	216 ± 12 ^b^
Glutamine	12,374 ± 279	13,849 ± 451	15,137 ± 835 ^a^	11,887 ± 357	11,962 ± 529 ^b^	13,656 ± 334
Alanine	3293 ± 99	3920 ± 151 ^a^	3972 ± 204 ^a^	3633 ± 131	3558 ± 132	3854 ± 112
*M. ext.digitorum longus*						
Valine	268 ± 9	552 ± 28 ^a^	177 ± 7 ^a^	336 ± 23 ^b^	339 ± 8 ^b^	330 ± 13 ^b^
Isoleucine	124 ± 4	235 ± 12 ^a^	82 ± 7 ^a,c^	199 ± 12 ^b^	194 ± 10 ^b^	182 ± 13 ^b^
Leucine	201 ± 8	354 ± 23 ^a^	558 ± 27 ^a,c^	297 ± 18 ^b^	325 ± 38 ^b^	297 ± 10 ^b^
Glutamine	8413 ± 322	9216 ± 293	9935 ± 480 ^a^	5880 ± 175 ^b^	6137 ± 193 ^b^	6838 ± 140 ^b^
Alanine	3499 ± 136	4642 ± 111 ^a^	5272 ± 235 ^a,c^	3635 ± 143	3762 ± 115 ^b^	3818 ± 110 ^b^

Means ± SE, *p* ˂ 0.05

^a^ compared to the corresponding control (SLD or SLD + S)

^b^ compared to the corresponding fed group

^c^ HLD (HLD + S) vs. HVLID (HVLID + S). Abbreviations as indicated in Table 1

**Table 5:** Amino acid concentrations in liver, jejunum, and kidneys (μmol/l of intracellular water)

	Fed animals			Overnight starved animals		
	SLD (*n* = 10)	HVLID (*n* = 10)	HLD (*n* = 9)	SLD + S (*n* =10)	HVLID + S (*n* = 10)	HLD + S (*n* = 10)
*Live*						
Valine	430 ± 26	946 ± 51 ^a^	438 ± 26 ^c^	449 ± 31	469 ± 32 ^b^	514 ± 25
Isoleucine	692 ± 65	555 ± 33 ^a^	321 ± 13 ^a,c^	308 ± 27 ^b^	280 ± 19 ^b^	307 ± 18
Leucine	603 ± 57	861 ± 50	1663 ± 158 ^a^	545 ± 35	534 ± 38 ^b,c^	580 ± 32 ^b^
Glutamine	15,588 ± 308	15,253 ± 646	14,247 ± 749	15,644 ± 1006	16,574 ± 1052	18,694 ± 639 ^a,b^
Alanine	6927 ± 340	6778 ± 321	6618 ± 335	3096 ± 378 ^b^	3371 ± 362 ^b^	4973 ± 224 ^a,b,c^
*Jejunum*						
Valine	725 ± 75	1528 ± 195 ^a^	918 ± 53 ^c^	632 ± 94	569 ± 48	562 ± 42
Isoleucine	477 ± 51	990 ± 146 ^a^	634 ± 47 ^c^	406 ± 61	357 ± 34	357 ± 30
Leucine	790 ± 73	1589 ± 215	3367 ± 644 ^a,c^	787 ± 109	623 ± 57	718 ± 48
Glutamine	1900 ± 202	1721 ± 148	1871 ± 149	1042 ± 79 ^b^	1358 ± 102	1456 ± 105
Alanine	6032 ± 431	6036 ± 233	7338 ± 249 ^a,c^	4107 ± 308	4495 ± 182	4,5003 ± 202
*Kidney*						
Valine	402 ± 33	675 ± 51 ^a^	410 ± 34 ^c^	404 ± 21	444 ± 26	444 ± 21
Isoleucine	308 ± 35	344 ± 27	295 ± 51	222 ± 11	239 ± 15	241 ± 17
Leucine	481 ± 33	503 ± 37	911 ± 73 ^a,c^	481 ± 20	241 ± 80 ^b^	483 ± 28 ^b^
Glutamine	4335 ± 193	4051 ± 123	4110 ± 312	3233 ± 216 ^b^	3249 ± 197	3324 ± 133 ^b^
Alanine	2156 ± 84	2331 ± 132	2801 ± 113 ^a,c^	1719 ± 81 ^b^	1857 ± 63 ^b^	2109 ± 64 ^b^

Means ± SE, *p* ˂ 0.05

^a^ compared to the corresponding control (SLD or SLD + S)

^b^ compared to the corresponding fed group

^c^ HLD (HLD + S) vs. HVLID (HVLID + S). Abbreviations as indicated in Table 1

### Effects of HVLID and HLD on glutamine synthetase and alanine aminotransferase activities

**Fig. 4:**
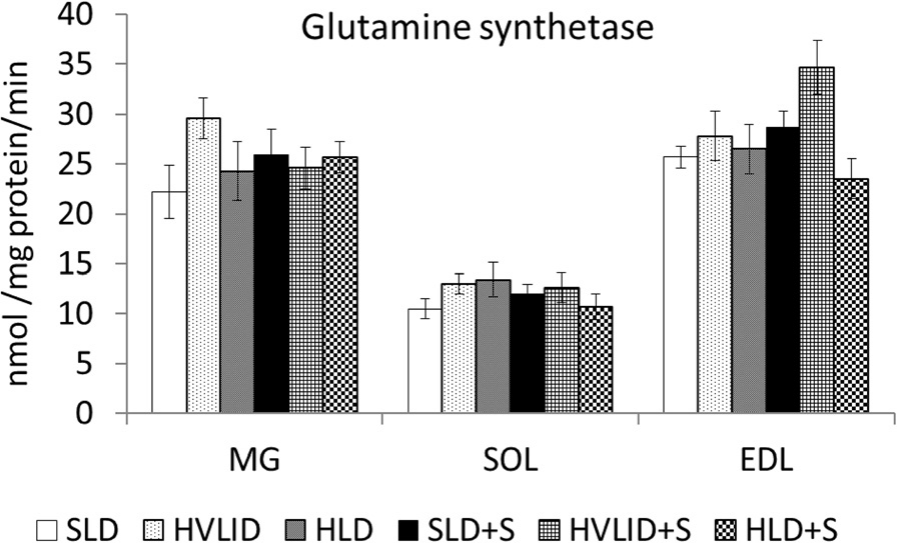
Glutamine synthetase. Means ± SE, *p* ˂ 0.05. *compared to the corresponding control (SLD or SLD + S); ^#^ compared to the corresponding fed group; ^†^ HLD (HLD + S) group vs. HVLID (HVLID + S) group

**Fig. 5:**
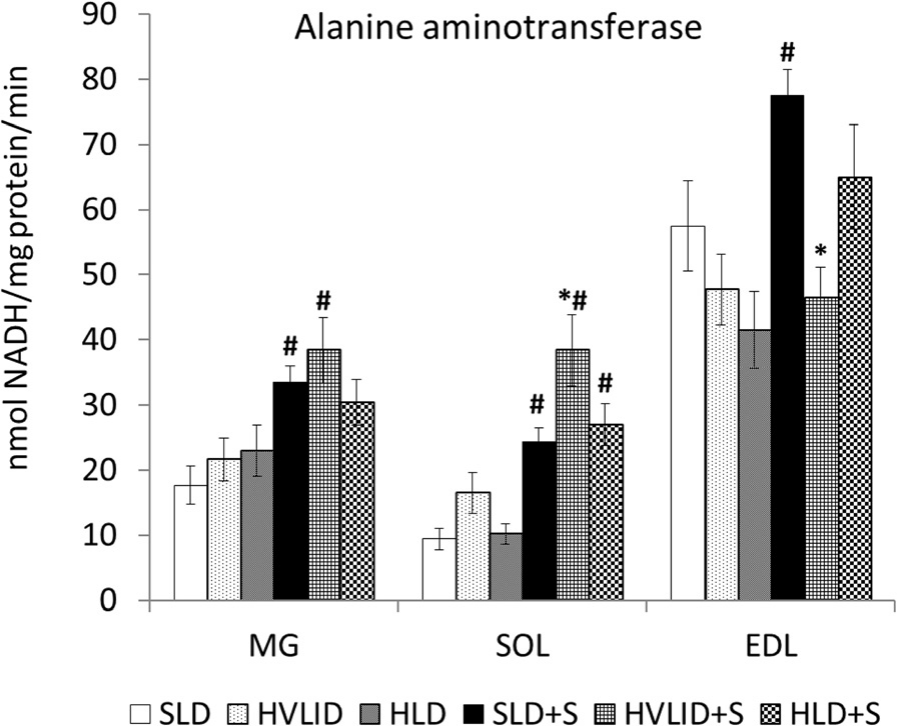
Alanine aminotransferase. Means ± SE, *p* ˂ 0.05. *compared to the corresponding control (SLD or SLD + S); ^#^ compared to the corresponding fed group; ^†^ HLD (HLD + S) group vs. HVLID (HVLID + S) group

The effect of chronic HVLID or HLD consumption on glutamine synthetase and alanine aminotransferase in muscles was not significant. Overnight starvation had no effect on glutamine synthetase, but a significant increase in alanine aminotransferase activity in muscles was found in the SOL and MG of animals fed various diets and the EDL of animals fed SLD before an overnight fast (Figs. 4 and 5).

### Effects of HVLID and HLD on protein synthesis

**Fig. 6:**
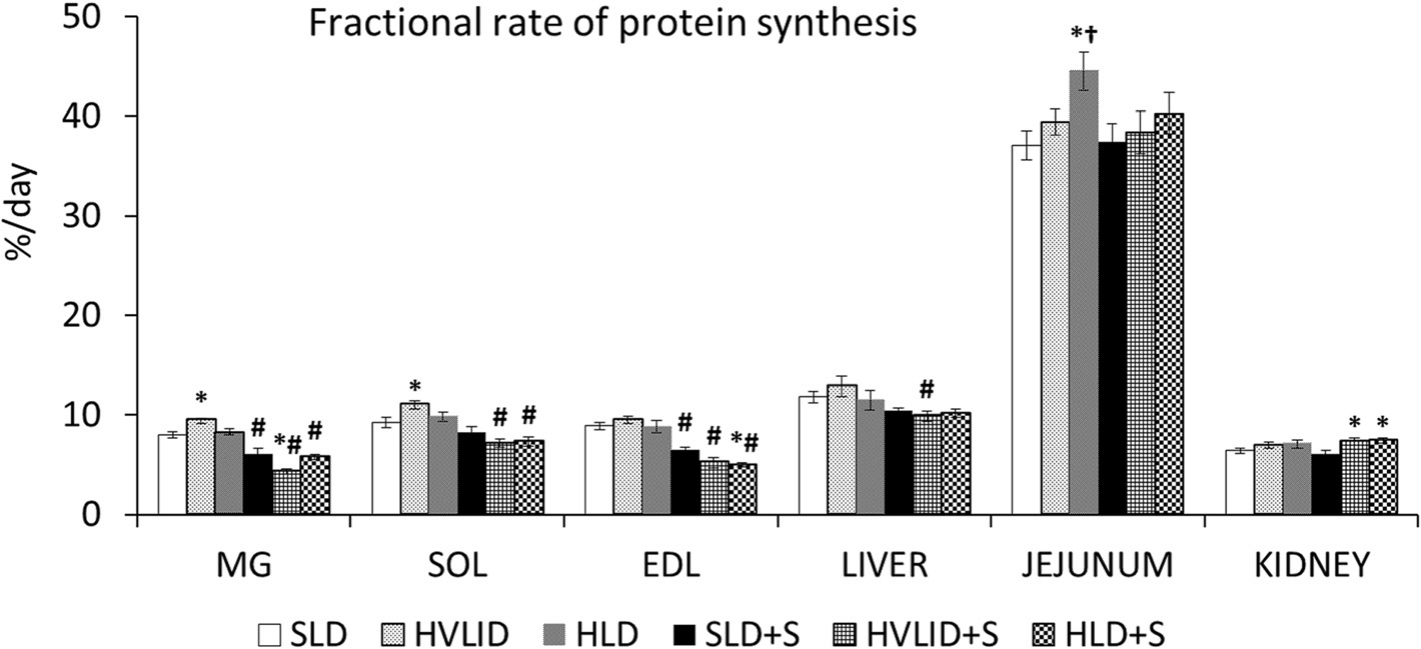
Fractional rate of protein synthesis. Means ± SE, *p* ˂ 0.05. *compared to the corresponding control (SLD or SLD + S); ^#^ compared to the corresponding fed group; ^†^ HLD (HLD + S) group vs. HVLID (HVLID + S) group

The fractional rate of protein synthesis (Fig. 6) was higher in the postprandial state in MG and SOL of animals fed an HVLID and in the jejunum of animals fed an HLD. A decrease in protein synthesis was found in muscles after an overnight fast. The decrease was more pronounced in gastrocnemius muscles of animals that consumed HVLID and in EDL muscles of animals that consumed HLD. Higher values of protein synthesis were observed in the postabsorptive state in the kidneys of HVLID and HLD groups compared to controls.

### Effects of HVLID and HLD on protein breakdown

**Fig. 7:**
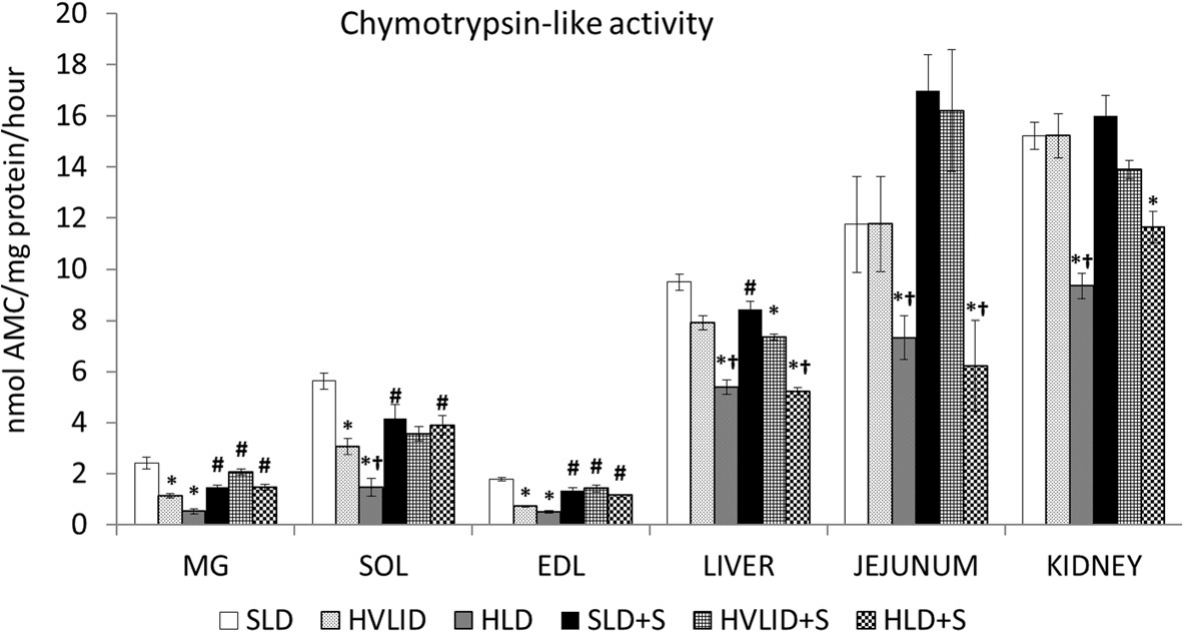
Chymotrypsin-like activity. Means ± SE, *p* ˂ 0.05. *compared to the corresponding control (SLD or SLD + S); ^#^compared to the corresponding fed group; ^†^ HLD (HLD + S) group vs. HVLID (HVLID + S) group

Consumption of HVLID decreased CHTLA activities in the muscles; HLD decreased CHTLA activities in the muscles, liver, jejunum, and kidneys (Fig. 7). Cathepsin B and L activities (Table 6) increased in the jejunum of the HVLID group. Lower activities were found in the SOL and jejunum of animals that consumed HLD compared to animals fed by HVLID. Effect of HVLID and HLD on CHTLA in muscles in postabsorptive state was insignificant. Cathepsin B and L activities in the jejunum and EDL of HVLID and HLD groups were lower than controls fed SLD before an overnight fast.

**Table 6:** Cathepsin B and L activities (nmol AMC/mg protein/hour)

	Fed animals			Overnight starved animals		
	SLD (*n* = 10)	HVLID (*n* = 10)	HLD (*n* = 9)	SLD + S (*n* =10)	HVLID + S (*n* = 10)	HLD (*n* = 10)
M. gastrocnemius	5.4 ± 1.1	4.3 ± 1.6	5.7 ± 1.2	6.5 ± 1.4	6.0 ± 0.9	5.9 ± 1.1
M. soleus	44.3 ± 2.6	51.4 ± 3.2	39.0 ± 2.1 ^c^	55.4 ± 3.8 ^b^	45.4 ± 1.6	27.4 ± 2.1 ^a,b,c^
M. ext. digitorum longus	12.6 ± 1.4	11.5 ± 1.4	8.0 ± 1.4	10.1 ± 4.7	4.7 ± 1.6 ^a,b^	1.6 ± 1.0 ^a,b^
Liver	213 ± 10	233 ± 11	192 ± 12	346 ± 21 ^b^	394 ± 14 ^b^	372 ± 16 ^b^
Jejunum	164 ± 7	214 ± 17 ^a^	98 ± 24 ^c^	270 ± 20	104 ± 16 ^a,b^	99 ± 23 ^a^
Kidney	1420 ± 47	1337 ± 96	831 ± 56	1566 ± 50	1126 ± 56	1021 ± 57

Means ± SE, *p* ˂ 0.05

^a^ compared to the corresponding control (SLD or SLD + S)

^b^ compared to the corresponding fed group

^c^ HLD (HLD + S) vs. HVLID (HVLID + S). Abbreviations as indicated in Table 1

### Effects of HVLID and HLD on weight and protein content of tissues

The effect of HVLID on muscle weight and protein content was not significant, but lower weight and protein content was found in EDL of animals that consumed HLD. Chronic intake of HVLID and HLD significantly increased the weight and protein content of the kidneys. We also observed higher liver weights in HLD fed animals (Table 7).

**Table 7:** Tissue weights, protein concentration, and protein content.

	Fed animals			Overnight starved animals		
	SLD (*n* = 10)	HVLID (*n* = 10)	HLD (*n* = 9)	SLD + S (*n* =10)	HVLID + S (*n* = 10)	HLD + S (*n* = 10)
*M. gastrocnemius*						
- Weight (g/kg b.w.)	4.16 ± 0.20	4.45 ± 0.21	4.09 ± 0.23	4.67 ± 0.67	4.69 ± 0.12	4.29 ± 0.14
- Protein (mg/g)	240 ± 8	234 ± 8	231 ± 5	233 ± 6	245 ± 7	238 ± 6
- Protein (mg/kg b.w.)	996 ± 58	1047 ± 72	946 ± 56	1088 ± 56	1144 ± 30	1027 ± 57
*M. soleus*						
- Weight (g/kg b.w.)	0.43 ± 0.01	0.48 ± 0.02	0.40 ± 0.01 ^c^	0.49 ± 0.01 ^b^	0.51 ± 0.01	0.42 ± 0.01 ^a,c^
- Protein (mg/g)	223 ± 14	218 ± 7	228 ± 11	225 ± 4	241 ± 5	243 ± 10
- Protein (mg/kg b.w.)	97 ± 3	104 ± 4	91 ± 6	110 ± 3	122 ± 5 ^b^	102 ± 6 ^c^
*M. ext. digitorum longus*						
- Weight (g/kg b.w.)	0.42 ± 0.01	0.43 ± 0.01	0.39 ± 0.00 ^a,c^	0.45 ± 0.01	0.45 ± 0.01	0.41 ± 0.01 ^a,c^
- Protein (mg/g)	271 ± 7	251 ± 6	272 ± 7	279 ± 8	262 ± 11	266 ± 8
- Protein (mg/kg b.w.)	114 ± 5	109 ± 4	97 ± 3 ^a^	127 ± 4	118 ± 5	109 ± 3 ^a^
*Liver*						
- Weight (g/kg b.w.)	35.87 ± 1.05	37.35 ± 0.68	39.35 ± 0.80 ^a^	27.36 ± 0.49 ^b^	26.96 ± 0.64 ^b^	30.07 ± 0.7 ^b,c^
- Protein (mg/g)	244 ± 8	242 ± 6	240 ± 8	320 ± 9 ^b^	270 ± 6 ^a,b^	278 ± 8 ^a,b^
- Protein (mg/kg b.w.)	8780 ± 462	9010 ± 235	9434 ± 386	8718 ± 190	7272 ± 184 ^b,c^	8350 ± 293
*Jejunum*						
- Protein (mg/g)	197 ± 7	181 ± 8	176 ± 5	199 ± 9	179 ± 12	166 ± 6
*Kidney*						
- Weight (g/kg b.w.)	3.21 ± 0.13	3.56 ± 0.07 ^a^	3.61 ± 0.10 ^a^	3.18 ± 0.07	3.53 ± 0.09 ^a^	3.44 ± 0.07 ^a^
- Protein (mg/g)	222 ± 8	229 ± 9	222 ± 4	213 ± 7	221 ± 5	222 ± 6
- Protein (mg/kg b.w.)	709 ± 28	815 ± 32 ^a^	798 ± 19 ^a^	674 ± 24	774 ± 9 ^a^	761 ± 18 ^a^

Means ± SE, *p* ˂ 0.05

^a^ compared to the corresponding control (SLD or SLD + S)

^b^ compared to the corresponding fed group

^c^ HLD (HLD + S) vs. HVLID (HVLID + S). Abbreviations as indicated in Table 1

## Discussion

Only moderate changes in food intake and body weight gain were observed between animals that consumed various diets in the initial phase of our study. Therefore, alterations in food intake did not affect our findings, but food composition, i.e., the replacement of 10 % of the basal diet with BCAAs or leucine alone exerted significant effects.

### Effects on amino acid concentration and metabolism

It is well established that the rate of BCAA degradation in skeletal muscle is highly responsive to changes in dietary intake. The K_m_ of BCAA aminotransferases for BCAAs is two- to four-fold higher than tissue BCAA concentrations [29]. Therefore, the rate of transamination leading to the production of glutamate and BCKA responds rapidly to changes in BCAA level. BCKA dehydrogenase activity in skeletal muscle is low, and most BCKA that is produced in muscle is released to the circulation and utilised in other tissues, especially the liver. The rise in plasma levels of all three BCKAs and ketoisocaproate in animals fed HVLID and HLD, respectively, indicates an enhanced load of these keto-acids in visceral tissues with unknown consequences. Enhanced KIC production from leucine may activate alternative pathways of KIC (ketoleucine) catabolism by the cytosolic enzyme KIC-dioxygenase in the liver. The result is an enhanced synthesis of ß-hydroxy-ß-methylbutyrate, which may be involved in the observed decrease in LDL cholesterol and muscle protein breakdown in animals fed HLD [30, 31].

To determine whether adaptive changes in enzyme activities in muscle tissue were involved in the rise in alanine and glutamine in blood plasma, alanine aminotransferase and glutamine synthetase activities were measured in muscles. Although marked alterations have been reported in various conditions, such dexamethasone treatment [27], chronic consumption of HVLID and HLD was without notable adaptive changes in these enzymes. Therefore, the main mechanism leading to enhanced alanine and glutamine production in skeletal muscle should be an increased flux of glutamate through glutamine synthetase and alanine aminotransferase resulting from enhanced glutamate production by BCAA aminotransferase.

A chronically enhanced release of BCKA, alanine, and glutamine from muscle may be related to the observed hypertrophy of the kidneys. Enhanced glutamine load may activate ammonia production in various tissues, especially gut and kidneys, and in the case of impaired ammonia detoxification to urea in liver disease induce symptoms of hepatic encephalopathy [32]. Enhanced ammonia concentrations in blood after BCAA supplementation were found during exercise and following a leucine intake >500 mg⋅kg^-1^⋅d^-1^ [33, 34]. Nevertheless, alterations in aminoacidemia induced by BCAA intake may also have favourable effects. The example might be positive influence of glutamine on the immune system, protein balance, and gut integrity.

The drop in concentrations of valine and isoleucine in blood plasma and muscles in animals fed high amounts of leucine is likely due to the well-known phenomenon that is referred to as BCAA antagonism [35]. Leucine stimulates the BCKA dehydrogenase complex that controls the rate limiting step in BCAA catabolism leading to enhanced catabolism of all three BCAAs. Depletion of valine and isoleucine pools may also be due to their competition with leucine for transport via the L-carrier system.

### Effects on protein metabolism in skeletal muscle

Higher protein synthesis rates and decreased CHTLA in the postprandial state in the muscles of animals fed an HVLID are consistent with most in vitro studies and/or studies that assessed the immediate response to BCAA administration [2, 36–38]. Therefore, our results confirm the regulatory effects of BCAAs on protein turnover, which may exert a positive influence on muscle protein balance. The marked decrease in CHTLA in muscles of animals fed an HLD is consistent with studies that concluded that leucine administration may specifically induce a reduction in protein breakdown without increasing protein synthesis [39–41]. However, we failed to demonstrate a positive effect of BCAA on muscle protein balance and a negative effect in EDL muscles exerted chronic consumption of excessive amounts of leucine. This was indicated by insignificant changes in weight and protein content in muscles of animals consuming HVLID and the decrease in EDL muscles in the HLD group.

We suppose that the discrepancy between the positive effects of HVLID on protein synthesis and proteolysis and HLD on proteolysis and the insignificant changes in muscle protein content may be explained by metabolic alterations in the postabsorptive state that were induced in our study by an overnight fast. Decreased rates of protein synthesis and CHTLA activities in overnight-fasted animals fed a normal diet represent metabolic adaptation that spares muscle protein in starvation. The more pronounced decrease in protein synthesis gastrocnemius muscles in HVLID group and in EDL muscles in HLD group of overnight-starved animals indicate an impaired metabolic response that is clearly not beneficial for muscle protein balance.

The adverse effect of a leucine-enriched diet is likely related to decreased intracellular pools of valine and isoleucine and preferential leucine oxidation. Both valine and isoleucine catabolites may be utilised in the citric cycle and promote consumption of pyruvate from glycolysis while the end product of leucine is acetyl Co-A, which blocks the entry of pyruvate into the citric cycle. Therefore, preferential leucine oxidation may exert negative effect on glycolysis, ATP production, and muscle performance, and lead to elevations in alanine aminotransferase activities in blood plasma. Enhanced plasma alanine aminotransferase activity without alterations in other markers of hepatocellular damage was reported after dietary leucine excess in muscle disease patients and apparently healthy individuals [42–44].

The finding of more significant alterations in EDL muscles compared to SOL or gastrocnemius muscles presents additional evidence that muscles composed mostly of white, fast-twitch fibres are more sensitive to various physiological or pathological signals than muscles composed mostly of red, slow-twitch fibres [45, 46].

A number of variables can modify the response to starvation, e.g., duration of starvation, muscle type, and ageing. Combaret et al. [47] reported that the rates of proteasome-dependent proteolysis were 1.5–2 fold higher in muscles in postabsorptive state and that with aging tended to decrease in the postabsorptive state and increase in the postprandial state. A role may play alterations in insulin sensitivity, the BCKA dehydrogenase activity, and amino acid concentrations [35, 48]. It should be noted that only protein synthesis rates can be measured directly under in vivo conditions. Proteolysis can be estimated indirectly, e.g. by changes MuRF1 and MAFbx expression, rates of ubiquitination, and activities of enzymes of ubiquitin-proteasome system [49]. Therefore, the results of the present study cannot exclude significant effects of chronic consumption of HVLID or HLD on proteolysis in postabsorptive state in other experimental conditions.

## Conclusions

To the best of our knowledge, this report is the first study to assess the metabolic response to BCAA and leucine supplementation in postprandial and postabsorptive states. We conclude that the chronic intake of a BCAA- or leucine-enriched diet significantly affects whole body metabolism as demonstrated by alterations in urea, alanine aminotransferase, and LDL cholesterol in blood, protein synthesis and proteolysis in various tissues, amino acid concentrations in blood and tissues, and liver and kidney weights. However, the results failed to demonstrate positive effects of the chronic consumption of BCAA or leucine-enriched diets on protein balance in skeletal muscle.

The non-significant effect of HVLID and rather negative effect of HLD on protein balance may be explained by depletion of valine and isoleucine pools in animals fed by an HLD and an impaired response to short-term starvation, which was characterised by impaired synthesis of muscle proteins, particularly in muscles with high content of white, fast-twitch fibres. These findings explain the discrepancy between the protein anabolic effects of BCAA or leucine on muscles that were reported under in vitro conditions and/or shortly after BCAA intake [2, 36–38] and their reduced or lack of effects following chronic administration [15, 50–52].

It should be noted that the present study was performed in healthy individuals without the adverse effects of signals that occur in several disorders, which lead to muscle wasting, and without the stimulatory effect of exercise on signalling pathways that activate protein synthesis. Further research is needed into the effects of chronic BCAA and leucine consumption in muscle-wasting disorders, the elderly, and during endurance exercise.

## Abbreviations

AMC: 7-Amino-4-methylcoumarin
BCAA: Branched-chain amino acids (valine, leucine, and isoleucine)
BCKA: Branched-chain keto acids
CHTLA: Chymotrypsin-like activity
FRPS: Fractional rate of protein synthesis
HLD: High leucine diet
HLD + S: Rats fed HLD starved overnight before sacrifice
HVLID: Diet with high content of valine, leucine, and isoleucine
HVLID + S: Rats fed HVLID starved overnight before sacrifice
KIC: α-Ketoisocaproate (ketoleucine)
KMV: α-Keto-β-methylvalerate (ketoisoleucine)
KIV: α-Ketoisovalerate (ketovaline)
SLD: Standard laboratory diet
SLD + S: Rats fed SLD starved overnight before sacrifice
